# Effect of Anti-Diabetic Medications on Dental Implants: A Scoping Review of Animal Studies and Their Relevance to Humans

**DOI:** 10.3390/ph15121518

**Published:** 2022-12-05

**Authors:** Sze Jun Tan, Badiah Baharin, Nurulhuda Mohd, Syed Nabil

**Affiliations:** 1 KPJ Ambulatory Care Centre Kinrara, 33, 35 & 37, Jalan BK 5a/2, Bandar Kinrara, Puchong 47180, Malaysia; 2 Department of Restorative Dentistry, Faculty of Dentistry, Universiti Kebangsaan Malaysia, Jalan Raja Muda Abdul Aziz, Kuala Lumpur 50300, Malaysia; 3 Department of Oral and Maxillofacial Surgery, Faculty of Dentistry, Universiti Kebangsaan Malaysia, Jalan Raja Muda Abdul Aziz, Kuala Lumpur 50300, Malaysia

**Keywords:** pre-clinical studies, animal models, hypoglycemic agents, osseointegration, diabetes mellitus, insulin

## Abstract

Animal studies have ascertained that hyperglycemia adversely affects bone metabolism and dental implant osseointegration. However, diabetic patients show low occurrence of unfavorable hard or soft peri-implant tissue changes, differences that are possibly due to treatment with anti-diabetic medications. This scoping review aimed to systematically examine the effects of these drugs on implant outcomes and explore the predictive modality of animal studies for clinical practice according to type 1 diabetes mellitus (T1DM) and type 2 diabetes mellitus (T2DM). Three electronic databases (MEDLINE, EBSCOHost, and Cochrane) were searched according to the PRISMA-ScR standards for studies on diabetic animals that received titanium implants and anti-diabetic treatments. Risk assessment was performed using the SYRCLE Risk-of-Bias (RoB) tool. Twenty-one papers were included, encompassing six types of medications. Fifteen studies were on T1DM animals, and only six involved T2DM models. T1DM animals were treated with non-insulin drugs in four investigations, while insulin was utilized in 11 other studies. In T2DM experiments, five administered non-insulin drugs, and only one applied locally delivered insulin. Only insulin in T1DM studies produced a positive influence on bone-implant contact (BIC), bone mineral content, and removal torque values. Inappropriate drug selection, inadequate glycemic control, and high RoB depict a mismatch between the research focus and the translational rationale to clinical practice. There remains a knowledge gap regarding T2DM investigations due to the lack of studies. More data are needed concerning intraoral implants and the performance of osseointegrated implants in patients with a later onset of diabetes. Future research should reflect the pathophysiology and treatment of each type of diabetes to ensure clinical applicability.

## 1. Introduction

Diabetes mellitus (DM) is a common non-communicable disease widely regarded as a relative risk factor in dental implant therapy [1]. It is characterized by persistent hyperglycemia resulting in micro- and macrovascular complications, namely, nephropathy, neuropathy, retinopathy, and cerebrovascular, coronary, and peripheral artery diseases. Type-1 diabetes mellitus (T1DM) and type-2 diabetes mellitus (T2DM) are the most common forms. Complete insulin deficiency occurs due to auto-immune destruction of pancreatic β-cell islets in T1DM. Conversely, resistance of body cells to insulin action leads to progressive reduction of its secretion and thus relative insulin deficiency in T2DM. Among all the types of diabetes, T2DM accounts for 90–95% of cases worldwide [2,3]. 

Besides the well-known systemic complications, hyperglycemia exerts generally deleterious effects on bone metabolism [1]. Reviews have detailed the findings from various animal models (murine, canine, swine, and rabbit) [4,5], revealing qualitative and quantitative impairment of the bone healing response to implant placement. In addition, histomorphometric and immunochemical analyses have consistently suggested compromised osseointegration and increased risk of implant failure in a high-glucose environment [1,6]. This evidence has led to a wide-ranging research effort to improve the outcomes and predictability of implant placement in diabetic conditions. 

Interestingly, clinical studies on implant outcomes in humans with DM have shown largely favorable results with high survival rates and a low incidence of complications or adverse soft or hard tissue changes [7]. Although osseointegration may be prolonged, it nonetheless occurs by 6 months, despite varying degrees of glycemic control [8]. One of the reasons for contradictory findings in experimental and clinical studies lies in the management of DM in humans. A diabetic patient typically undergoes glucose monitoring using glycated hemoglobin (HbA1c), dietary counseling, and weight management and receives various anti-diabetic medications ranging from oral hypoglycemic agents (OHA) to insulin injections. Reducing blood glucose levels by these means reverses cellular dysfunction and immune and inflammatory defects, thereby minimizing disease complications [9].

A crucial metabolic difference in the pathophysiology of T1DM and T2DM guides the pharmacological management of this disease. The treatment of DM, which includes both oral and injectable medications, emphasizes achieving normoglycemia and maintaining good glycemic control to prevent complications [9]. Insulin replacement therapy is mandatory for the complete absence of this hormone in T1DM patients [4]. T2DM, which presents with hyperinsulinemia at an early stage, is treated by OHAs aimed at promoting insulin sensitivity [10], preventing glucose absorption in the intestines, and stabilizing post-prandial glucose levels [11]. As the disease progresses, a gradual reduction in pancreatic β-cells leads to depletion of insulin in T2DM, and at this stage, patients require insulin injection. Studies have indicated that OHAs [12] and insulin [4] have anabolic and anti-resorptive effects on bone metabolism, thereby potentially promoting osseointegration. Although there are several reviews on implant osseointegration in hyperglycemic animals [4,13], none have elucidated the effects of individual anti-diabetic medications in relation to the different types of diabetes.

Ideally, animal research should reflect the specific underpinnings of DM pathophysiology and therapy in humans. Therefore, this scoping review aims to systematically chart the extant research done in this area and identify gaps in knowledge regarding the effects of anti-diabetic drugs on implant outcomes in T1DM and T2DM animals. Correspondingly, the relevance of these diabetic animal studies to clinical practice in implant therapy will be discussed.

## 2. Results

### 2.1. Study Selection

**Figure 1:**
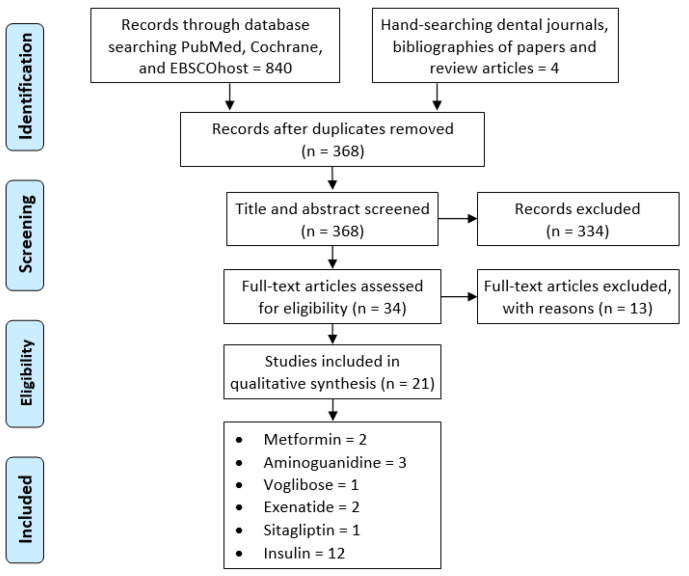
Flowchart of review process according to the Preferred Reporting Items for Systematic Reviews and Meta-Analyses extension for Scoping Reviews (PRISMA-ScR) guidelines.

A total of 840 articles was identified from the initial database search. An additional four studies were retrieved from hand-searching the references of reviews and collected records. After duplicate removal, 368 titles/abstracts were carefully screened, and 34 full-text articles were found to be relevant. Thirteen papers were excluded for the following reasons: the use of supplements or off-label drugs for diabetes treatment (*n* = 6) [14,15,16,17,18,19], no treatment for diabetic animals (*n* = 3) [20,21,22], anti-diabetic drug given to healthy animals (*n* = 1) [23], the use of a non-diabetic animal model (osteoporosis) (*n* = 1) [10], no titanium implants placed (*n* = 1) [24], and less than 10 implants in the study design (*n* = 1) [25] (Supplement C). A final count of 21 publications that fulfilled the inclusion and exclusion criteria was included in this review (Figure 1).

### 2.2. Risk of Bias (RoB) Assessment 

Table 1 summarizes the RoB assessment of all selected studies using the recommended SYRCLE tool (Supplement D). Only 4 out of 21 reports [26,27,28,29] had a low RoB, while all others were considered unclear or high risk. The domains of selection, performance, and detection biases were distinctly lacking, where very few authors reported on sequence allocation procedures, animal randomization and its methods, and blinding of investigators and outcome assessors. In addition, the use of animal research reporting guidelines (e.g., the Animal Research: Reporting of In Vivo Experiments (ARRIVE) checklist [30]) was found to be wholly absent apart from one study [29].

### 2.3. Characteristics of the Included Studies

The characteristics of the included studies are presented in Table 2. All experiments were carried out in a variety of young male rats except for a study performed in rabbits [37]. Less than a third of the studies were on T2DM animals. Among T1DM models, the induction methods ranged from 30 to 80 mg/kg streptozocin (STZ) injection or 42 to 115 mg/kg alloxan. DM was mostly induced at the beginning of the study, but anti-diabetic drugs were administered before, during, or even up to two weeks after implant placement. Interestingly, four studies [27,28,29,36] had diabetic induction after 4 to 8 weeks of implant osseointegration. 

The included publications used insulin and five non-insulin drugs, namely, aminoguanidine, sitagliptin, metformin, voglibose, and exenatide. Four experiments in T1DM models utilized non-insulin drugs as the treatment modality [26,31,32,33]. One of the six T2DM studies assessed the effects of locally administered insulin on osseointegration [47]. The main outcomes of interest were the percentages of bone-implant contact (BIC) and bone volume (BV), as well as counter-torque values (N/cm), as progress indicators of implant osseointegration. Table 3 details the location of fixture placement, drug type, and its ability to control the level of glycemia, as well as the implant outcomes in each included study.

### 2.4. Effects of Anti-Diabetic Drugs on Implant Outcomes in T1DM Animal Models

#### 2.4.1. Aminoguanidine

Two investigations analyzed the intraperitoneal administration of this hydrazine-derived drug [26,31], while another experimented with local placement of an aminoguanidine-loaded membrane [32] at the site of implant installation in T1DM. Kopman et al. [26] observed a significant increase in BIC values akin to healthy controls when this medication was administered. Similarly, Aiala et al. [32] and Guimarães et al. [31] described comparable counter-torque values in healthy Wistar and diabetic rats treated with aminoguanidine. All three analyses corroborate significantly reduced osseointegration in untreated diabetic animals. Notably, the animals in these three experiments remained hyperglycemic throughout the study because aminoguanidine does not affect glucose metabolism.

#### 2.4.2. Sitagliptin

A study by Bautista et al. [33] noted statistically lower implant bone area fraction occupancy (BAFO) in diabetic rats compared with their normal counterparts. However, administration of oral sitagliptin not only failed to reduce the blood glucose level, but there was also no influence of the drug on the measured parameters and bone microarchitecture examined. The author pointed out that this could be attributed to the method of DM induction (10% fructose consumption and a low dose of STZ injection (40 mg/kg)), resulting in T1DM animals instead of the intended T2DM model. Furthermore, because sitagliptin acts as a stimulator of insulin secretion, the glucose-lowering effect is not produced, as there is already widespread destruction of pancreatic β-cells required for insulin production typical of T1DM. Hence, the investigators surmised that the continued hyperglycemic state may preclude the reversal of the negative effects of diabetes by sitagliptin.

#### 2.4.3. Insulin

Six of the studies utilizing systemic insulin treatment for glucose control in T1DM rats found significantly improved BIC compared with untreated animals, indicating enhancement of osseointegration [27,34,35,36,38,39]. New bone area was reported to return to normal levels in diabetic rats after insulin therapy, suggesting the regulatory role of this hormone in bone metabolism and remodeling, apart from glucose control [38]. Quantitative subtraction imaging revealed greater peri-implant bone density and higher mineral content with insulin [28]. Mechanical retention was also improved, as demonstrated by higher removal torque values akin to healthy animals [27,35,37,41]. Conversely, diabetic rats without insulin control demonstrated gradual loss of implant-to-bone integration, denoted by decreasing BIC values [36]. This finding indicates that continual insulin therapy is also crucial for long-term maintenance of osseointegration after implant placement. 

In contrast, McCracken et al. [40] found higher peri-implant bone production in STZ-induced diabetic rats compared with healthy animals, and insulin treatment conflictingly lowered the total bone volume around implants by 24 days of healing. The authors suggested that the findings could be due to obtunded bone remodeling and decreased osteoclastic activity in diabetic animals, resulting in higher bone volume than healthy controls. This gives rise to some uncertainty about the positive effects of insulin postulated by other studies, and insulin may not entirely reverse the adverse effects of hyperglycemia on bone growth and metabolism [48]. 

One study used locally delivered, insulin-containing, sustained-release vehicles for peri-implant healing in T1DM rats [41]. There was significantly improved biomechanical retention of implants by local insulin application. However, no actual treatment was provided for the DM, and systemic hyperglycemia was maintained throughout the study. 

### 2.5. Effects of Anti-Diabetic Drugs on Implant Outcomes in T2DM Animal Models

#### 2.5.1. Biguanide Drug—Metformin

Two studies [42,43] utilizing T2DM rodent models failed to draw firm conclusions on the effect of metformin on osseointegration. BIC in Goto–Kakizaki (GK) rats treated with metformin [43] showed encouraging results at week 1. However, the significance was lost by the fourth week of post-implant healing. The authors attributed this outcome to fluctuation in blood glucose levels to hyperglycemia at 4 weeks, even with metformin administration. Serrão et al. [42] found no difference in BIC and bone area (BA) between groups of Wistar rats when metformin was given as anti-diabetic treatment, despite controlling blood glucose levels. However, the expression of bone-promoting osteoprotegerin (OPG) was increased, and the receptor activator of nuclear factor kappa-Β ligand (RANKL) to OPG ratio was reduced in peri-implant medullary bone. This may indicate the possible benefits of metformin on a molecular level [42]. 

#### 2.5.2. Alpha-Glucosidase Inhibitor—Voglibose 

A publication by Hashiguchi et al. [44] reported no improvement in BIC and removal torque strength in GK rats despite administering voglibose. Moreover, the drug did not adequately regulate blood glucose levels, and the animals remained hyperglycemic throughout the study (glucose above 160 mmol/L). The authors concluded that anti-diabetic drugs cannot fully reverse the deleterious effect of hyperglycemia on bone metabolism and mineralization.

#### 2.5.3. Exenatide

Zhou and colleagues [46] described the usage of delayed-release exenatide microspheres before and during implant placement in Zucker diabetic fatty (ZDF) rats. The researchers noted that serum alkaline phosphatase (ALP), a biomarker of bone remodeling, was elevated in the exenatide-treated T2DM rats. A follow-up study by Liu et al. [45] demonstrated substantially increased integrin α5β1 and fibronectin expression on the surface of peri-implant osteoblasts in rats with constant glucose levels controlled by exenatide. Integrin α5β1 and fibronectin are extracellular peptides that promote osteoblastic attachment to the cementum or titanium surface, thereby enhancing osseointegration. Histologically, the exenatide-treated group with normal glucose had higher peri-implant bone density and more regularly arranged trabecular bone [45].

#### 2.5.4. Insulin

Only one study [47] described the use of locally released insulin at the site of implant–bone interface in T2DM GK rats. BIC at 6 weeks was significantly greater in the diabetic rats that received insulin-coated implants, compared with plain titanium implants in the comparison group. However, the percentage was still notably lower than the BIC in healthy Sprague–Dawley rats. The authors inferred that insulin independently increased osteoblastic proliferation and promoted new bone formation. Nevertheless, no systemic glucose control treatment was given, and the glycemic level of the GK rats during the implantation, healing, and euthanasia period was also not specified.

## 3. Discussion

It is axiomatic that well-designed animal research is essential to generate high-quality molecular and cellular levels of evidence that could be translated to improve clinical practice. This scoping search revealed that almost all existing research was conducted on T1DM models, as noted in a previous review [4], even though T2DM is far more prevalent in humans. This could be due to lower costs and easier mode of disease initiation. Numerous animal strains have been used, while the methods of DM induction, the anti-diabetic medication dosage and delivery, the timing of disease, and implant placement also differed markedly among the studies.

The blood glucose levels of both T1DM and T2DM animals varied widely from 200 to well above 500 mg/dL and either fluctuated [43,45] or remained hyperglycemic [26,32,33,38,44] throughout the study period, despite the administration of anti-diabetic medication. This experimental design mirrors the human condition of uncontrolled severe diabetes [4]. Failure to control the level of glycemia, deviation from the intended intervention [40], and lack of standardization have rendered the results of these animal studies inconsistent, biased, and thus unable to provide firm conclusions regarding the effect of anti-diabetic treatment on implant osseointegration. The RoB assessment also indicated an evident inadequacy in accounting for relevant experimental context. This too suggests a great need for authors to explicitly address blinding and randomization, heterogeneity of disease severity, husbandry, and reporting quality as vital parts of a good research design [49].

The majority of the studies was of short duration, ranging from 4 to 16 weeks. All but one investigation [37] utilized rat models. Rats have much higher bone metabolism and turnover rate than humans, which is favorable for experimental costs and animal management [50]. However, their small stature permits only limited types of surgeries and small adapted implants of 1 mm in width and 2 mm in length [51]. Consequently, the findings in small animal models often do not translate into human clinical application [52,53,54]. The current evidence base of rat experiments revealed a much greater percentage of studies employing long bone models (tibia or femur) compared with oral bone models (maxilla or mandible extraction sockets) [51]. This propensity is reflected in our review, where almost all studies placed the titanium implant at an extraoral long bone site. This relatively sterile setting is in stark contrast to the constant challenge of bacterial plaque and masticatory activity within the oral cavity. Intraoral titanium implants are always subject to the risk of osseointegrative complications and peri-implant disease in the presence of food, microorganisms, and functional load. This fundamental difference with the enclosed and protected healing environment of long bone sites could call into question the comparability with true clinical conditions [51]. In this regard, porcine models, such as Göttingen minipigs [55], may be a better representative model for human bone regeneration; they have remarkably similar physiology, metabolism, and pancreatic anatomy to humans. Furthermore, porcine bone homeostasis and structure, with their larger jawbone and blood volume, would allow for intraoral investigations using multiple dental implants and easy sampling for bone biomarkers and genetic studies [56]. 

Common anti-diabetic drugs such as biguanides, alpha-glucosidase inhibitors, glucagon-like peptide-1 (GLP-1) analogs, dipeptidyl peptidase-4 (DPP-4) inhibitors, and insulin were used in these animal experiments. The mechanism of action (MOA) of aminoguanidine in the treatment of DM is through scavenging free radical groups and inhibition of advanced glycation end products (AGEs) formation, thereby interrupting oxidative cycle damage and preventing AGE-related complications [31]. Sitagliptin is a selective DPP-4 inhibitor that blocks the inactivation of incretins (GLP-1 and gastric inhibitory polypeptides). Native insulin secretion is stimulated, and postprandial glucose elevation is stabilized, improving glycemic control. Metformin decreases gluconeogenesis and glucose absorption in the intestines while promoting peripheral insulin sensitivity [10]. Voglibose is a recently approved α-D-glucosidase inhibitor that competitively inhibits postprandial glucose absorption and delays the rise of blood glucose concentration [11]. Exenatide is a GLP-1 receptor agonist (GLP-1RA) that aids glycemic control by promoting insulin secretion and inhibiting glucagon action while delaying gastric emptying. These five non-insulin drugs are mainly employed to treat T2DM or are used in combination with insulin to manage T1DM.

Based on the above-mentioned studies, most non-insulin medications either failed to achieve acceptable glycemic control [26,32,43,44,45,46] or did not improve any measure of osseointegration [42,43]. Sitagliptin was inappropriately selected for T1DM rats, rendering the results doubtful and of little research value [33]. Aminoguanidine may enhance peri-implant bone healing in T1DM animals [26,31,32]. However, using this drug in clinical practice does not obviate the need for insulin for glycemic control in T1DM patients, as its MOA does not influence glucose levels [57]. Furthermore, this drug has been reported to cause significant side effects in humans, including elevation of liver enzymes, production of autoantibodies, vasculitis, and anemia, and possibly initiating pancreatic and kidney tumors [58]. Future studies must consider the pathophysiology of DM and drug MOA in their methodology to justify the use, efficacy, safety and impact of anti-diabetic drugs in implant osseointegration. 

Insulin is a peptide hormone that regulates blood glucose levels by stimulating glucose uptake into cells and conversion into glycogen for storage. This therapy appears to be the only anti-diabetic agent that shows a positive influence on glucose control, osseointegration, and reversal of bone changes [36] in T1DM compared with other medications. While T1DM patients are entirely dependent on insulin from a young age, treatment for T2DM typically shifts to insulin injections only at the later stages of the disease [2]. Thus, more research on orally administered drugs, including sulfonylureas and thiazolidinediones, could provide additional insight into implant osseointegration and bone metabolism in patients with T2DM. No extant study has yet analyzed the effect of systemic insulin in more mature T2DM animals, and comparability with the outcomes in T1DM remains unknown.

The present review noted three studies that utilized local drug delivery methods at the implant site to improve osseointegration [32,41,47]. While the results were largely favorable with higher BIC and counter-torque values, the local infiltration of anti-diabetic drugs had a limited time effect, and the animals remained systemically hyperglycemic. The brief study period could not demonstrate the benefits of such methods towards long-term maintenance of osseointegration after completion of drug release. The intricate and dynamic process of bone homeostasis does not halt after the formation of the bone–implant interface. Past evidence has highlighted that uncontrolled DM may lead to bone resorption, reversal of BIC, and possible loss of the osseointegrated implant [27,29,36]. New studies should focus on the potential of locally delivered drugs as an adjunct to systemic anti-diabetic medication, rather than as a mainstay approach, to elucidate whether an actual benefit reflecting human disease management could be obtained.

Despite the attenuated bone turnover and sluggish rate of bone formation, both animal [44,56] and clinical [8,59] studies generally concur that implant osseointegration appears to proceed continually despite the hyperglycemic state. In four notable studies [27,28,29,36], the researchers inserted implants in healthy animal models and allowed osseointegration to occur for up to 2 months prior to induction of DM. This scenario is remarkably analogous to human conditions, where patients with dental implants develop the systemic disease at a later age. These T1DM studies demonstrated that with the maintenance of normal blood glucose levels using systemic insulin therapy, the bone density, BIC, and counter-torque values remained comparable to the healthy controls. In contrast, animals without insulin treatment demonstrated loss of bone density and BIC. Therefore, this method is a plausible prospective research framework to address the loss of osseointegration of stable implants rather than acceleration of initial bone formation, providing data that is relevant to actual clinical practice and in patients with T2DM. 

The measures of osseointegration in humans are not limited to only BIC, bone volume, or removal torque, but rather non-invasive clinical observations such as the presence of bleeding on probing, peri-implant pocket depth, implant stability quotient (ISQ) values, and radiographic marginal bone loss. Additionally, longer-term implant survival after placement provides a better picture of an implant in function, especially when observing the effect of lifelong diseases such as DM on the outcome of implant therapy. The experiment by Yamazaki et al. [29] is the only animal study to have measured peri-implant clinical parameters in hyperglycemia. More of such evidence is necessary to emulate the complications of peri-implant disease in clinical practice.

Finally, this project has two main limitations. Firstly, as a scoping study, the focus is broad in nature, aimed at identifying the extent and features of existing research on this topic. Thus, the depth of the syntheses could be better developed in future systematic reviews. Secondly, this review is unable to contribute firm conclusions regarding the true effect of anti-diabetic drugs on implants in patients with DM due to the heterogenous nature of the data and syntheses. Despite these shortcomings, our approach and results serve to generate insight into where more research should be conducted and steer new experimental endeavors in a better direction that is more reflective of clinical practice.

## 4. Materials and Methods

This review was performed according to the Preferred Reporting Items for Systematic Reviews and Meta-Analyses extension for Scoping Reviews (PRISMA-ScR) [60] checklist (Supplement A). Three electronic databases were comprehensively searched (Cochrane Central Register of Controlled Trials (CENTRAL), MEDLINE/PubMed, and EBSCOhost) based on various combinations of the following terms: “dental implants”, “diabetes mellitus”, “animal”, “pre-clinical”, “experimental”, “hypoglycemic agents”, “osseointegration”, “titanium”, and “insulin” (Supplement B) for documents published up to May 2022. All quantitative animal studies that matched the PICO research framework—population of diabetic animals receiving titanium implants, intervention by treatment using approved human anti-diabetic medications [9], comparator using untreated diabetic animals, and outcomes of implant treatment (Table 4)—were included in this review. The types of sources involved prospective animal experiments with and without anti-diabetic treatment. A sample size of at least 10 implants was required, and no other restrictions were placed on the year of publication, language, or sources. Additional studies were gleaned from the bibliographies of all eligible papers from the electronic search. Clinical research on humans, review papers, case studies, and studies that did not provide diabetic treatment or utilized supplements or off-label drugs for DM were excluded. Articles that involved provision of anti-diabetic drugs to non-diabetic animals were also omitted.

The citations from the search were imported into Mendeley Reference Manager (Mendeley Ltd., London, UK), and duplicate entries were automatically removed. After further screening according to the titles and abstract, three reviewers (B.B., N.M., and S.J.T.) examined the full texts of the remaining articles. Studies that were irrelevant to the research topic were omitted. The Risk of Bias (RoB) tool by the Systematic Review Centre for Laboratory Animal Experimentation (SYRCLE) was employed to assess the bias level in each publication [61]. Any disagreements during study selection and bias evaluation were discussed between all three reviewers to reach a consensus. The findings of each eligible study were individually extracted into Microsoft Excel Spreadsheets (Microsoft Corp, Redmond, WA, USA) and analyzed according to the type of anti-diabetic medication. Finally, a descriptive synthesis regarding the effect of each drug on dental implant outcomes was undertaken.

## 5. Conclusions

Insulin administration in these short-term T1DM animal studies evidently improves BIC, implant retention, and other osseointegration measures. Furthermore, maintaining good glycemic control with insulin may preserve the success of osseointegrated implants, even with late induction of the systemic disease. Other OHAs and non-insulin drugs used in both T1DM and T2DM animal models produced an inconclusive benefit on implant osseointegration. The extant animal studies on the effect of anti-diabetic drugs on implant outcomes are currently of uncertain relevance to true human conditions. The lack of clinical applicability is due to the methodological errors of inappropriate drug/DM model selection, disparate disease induction methods and drug dosage/delivery regimens, and variable severity of glycemia despite drug administration. There is a definite research gap concerning T2DM investigations in larger animal models, intraoral implant placement, and the effect of more common systemic oral hypoglycemic drugs on implant outcomes. Finally, there is a considerable need for longer-term pre-clinical studies on the performance of the osseointegrated dental implant should a healthy patient develop diabetes at a later age.

## Figures and Tables

**Table 1:** Summary of the risk-of-bias according to the Systematic Review Centre for Laboratory Animal Experimentation (SYRCLE) RoB tool.

Author and Year	Signaling Questions *										Summary
	1	2	3	4	5	6	7	8	9	10	
Guimarães et al., 2011 [31]	+	?	−	−	−	−	−	+	+	?	?
Aiala et al., 2013 [32]	+	+	+	−	−	−	−	+	+	−	?
Kopman et al., 2005 [26]	+	+	?	−	−	+	+	+	+	−	+
Bautista et al., 2019 [33]	−	+	−	−	−	−	+	+	+	−	−
Matsubara et al., 2001 [34]	−	+	−	−	−	−	−	+	+	+	?
Zhang et al., 2021 [35]	+	+	?	−	−	−	−	+	+	+	?
de Molon et al., 2013 [27]	+	+	?	−	−	+	+	+	+	+	+
de Morais et al., 2009 [28]	+	+	?	−	−	+	+	+	+	+	+
Kwon et al., 2005 [36]	−	−	−	−	−	−	+	+	+	?	?
Margonar et al., 2003 [37]	+	+	?	−	−	−	−	+	+	+	?
Siqueira et al., 2003 [38]	−	+	−	−	−	−	−	+	+	−	−
Fiorellini et al., 1999 [39]	−	+	−	−	−	−	−	+	+	+	?
McCracken et al., 2006 [40]	−	+	−	−	−	−	−	−	+	−	−
Yamazaki et al., 2020 [29]	+	+	+	−	−	−	+	+	+	+	+
Han et al., 2012 [41]	−	+	−	−	−	−	−	+	+	−	−
Serrão et al., 2017 [42]	+	+	?	?	+	−	?	+	−	−	?
Inouye et al., 2014 [43]	−	−	−	−	−	−	−	+	+	−	−
Hashiguchi et al., 2014 [44]	−	+	−	−	−	−	−	+	+	−	−
Liu et al., 2015 [45]	−	+	−	−	−	−	−	+	+	?	?
Zhou et al., 2015 [46]	−	+	−	−	−	−	−	+	+	?	?
Wang et al., 2011 [47]	−	+	−	−	−	−	−	+	+	?	?

* Signaling questions: 1: Was the allocation sequence adequately generated and applied? 2: Were the groups similar at baseline or were they adjusted for confounders in the analysis? 3: Was the allocation adequately concealed? 4: Were the animals randomly housed during the experiment? 5: Were the caregivers and investigators blinded from the intervention given to each animal? 6: Were animals selected at random for outcome assessment? 7: Was the outcome assessor blinded? 8: Were incomplete outcome data adequately addressed? 9: Was the study free of selective outcome reporting? 10: Was the study apparently free of other problems that could result in high risk of bias? +: Low risk of bias; ?: Unclear risk; −: High risk of bias.

**Table 2:** Animal species/strain, demographics (age, sex), number of implants, timing and mode of induction, timing of anti-diabetic medication.

Author and Year	Animal Model	Sex	Age	Number of Implants	Type of DM and Medication	Induction of DM	Timing of Diabetes Induction	Timing of Anti-Diabetic Medication
Guimarães et al., 2011 [31]	Wistar rats	Not specified	20 weeks	6 per group, total 36	T1DM (aminoguanidine)	Intraperitoneal alloxan injection, 84 mg/kg	Before implant placement	Started the day of implant placement
Aiala et al., 2013 [32]	Wistar rats	Male	28 weeks	8 per group, total 48	T1DM (aminoguanidine)	70 mg/kg streptozocin	Before implant placement	Started the day of implant placement
Kopman et al., 2005 [26]	Sprague–Dawley rats	Male	Not specified	8 per group, total 32	T1DM (aminoguanidine)	70 mg/kg streptozocin	Before implant placement	Started the day of implant placement
Bautista et al., 2019 [33]	Wistar rats	Male	16 weeks	8 per group, total 32	T1DM (sitagliptin)	40 mg/kg streptozocin	Before implant placement	Started the day after implant placement
Matsubara et al., 2001 [34]	Wistar rats	Male	12 weeks	12 per group, total 36	T1DM (insulin)	60 mg/kg streptozocin	Before implant placement	Before implant placement
Zhang et al., 2021 [35]	Sprague–Dawley rats	Male	11 weeks	6 per group, total 30	T1DM (insulin)	30 mg/kg streptozocin	Before implant placement	Before implant placement
de Molon et al., 2013 [27]	Wistar rats	Male	16 weeks	20 per group, total 80	T1DM (insulin)	40 mg/kg streptozocin	2 months after implant placement	Day 2 of DM
de Morais et al., 2009 [28]	Wistar rats	Male	16 weeks	10 per group, total 40	T1DM (insulin)	40 mg/kg streptozocin	2 months after implant placement	Day 2 of DM
Kwon et al., 2005 [36]	Sprague–Dawley rats	Male	4 weeks	4 per group, total 32	T1DM (insulin)	70 mg/kg streptozocin	28 days after implant placement	28 days after implant placement
Margonar et al., 2003 [37]	New Zealand rabbits	Female	20 weeks	9 per group, total 27	T1DM (insulin)	Intraperitoneal alloxan injection 115 mg/kg	Before implant placement	Day 2 of DM
Siqueira et al., 2003 [38]	Wistar rats	Male	12 weeks	Control 17, diabetic 18, insulin 8, total 43	T1DM (insulin)	Intraperitoneal alloxan injection 42 mg/kg	Before implant placement	10 days after implant placement
Fiorellini et al., 1999 [39]	Sprague–Dawley rats	Male	5 weeks	5 per group, total 10	T1DM (insulin)	70 mg/kg streptozocin	Before implant placement	Day 2 of DM
McCracken et al., 2006 [40]	Sprague–Dawley rats	Not specified	Not specified	60 diabetic, 60 healthy, 32 insulin, total 152	T1DM (insulin)	60 mg/kg streptozocin	Before implant placement	Ongoing insulin treatment
Yamazaki et al., 2020 [29]	Wistar rats	Male	5 weeks	12 per group, total 36	T1DM (insulin)	50 mg/kg streptozocin	5 weeks after implant placement	Day 3 of DM
Han et al., 2012 [41]	Wistar rats	Male	Not specified	6 per group, total 48	T1DM (insulin)	80 mg/kg streptozocin	Before implant placement	Slow release insulin coated implant at placement
Serrão et al., 2017 [42]	Wistar rats	Male	12 weeks	10 per group, total 30	T2DM (metformin)	10% fructose and 40 mg/kg streptozocin	Implant placement in spontaneously diabetic animal	2 weeks after implant placement
Inouye et al., 2014 [43]	Goto–Kakizaki (GK) rats	Male	12 weeks	12 per group, total 36	T2DM (metformin)	Genetically modified, spontaneous T2DM	Implant placement in spontaneously diabetic animal	Ongoing metformin treatment
Hashiguchi et al., 2014 [44]	Goto–Kakizaki (GK) rats	Male	Not specified	10 per group, total 20	T2DM (voglibose)	Genetically modified, spontaneous T2DM	Implant placement in spontaneously diabetic animal	Ongoing voglibose treatment
Liu et al., 2015 [45]	Zucker diabetic fatty (ZDF) rats	Male	12 weeks	11 per group, total 33 rats, 66 implants	T2DM (exenatide)	Genetically modified, spontaneous T2DM	Implant placement in spontaneously diabetic animal	Ongoing subcutaneous injection
Zhou et al., 2015 [46]	Zucker diabetic fatty (ZDF) rats	Male	12 weeks	11 per group, total 33 rats, 66 implants	T2DM (exenatide)	Genetically modified, spontaneous T2DM	Implant placement in spontaneously diabetic animal	Ongoing subcutaneous injection
Wang et al., 2011 [47]	Goto–Kakizaki (GK) rats	Not specified	Not specified	10 per group, total 20, also 10 control Sprague–Dawley rats	T2DM (insulin)	Genetically modified, spontaneous T2DM	Implant placement in spontaneously diabetic animal	Slow release insulin coated implant at placement

T1DM: type 1 diabetes mellitus; T2DM: type 2 diabetes mellitus.

**Table 3:** Summary of findings (anti-diabetic drug used, location of implant, glucose control after medication, and outcomes of interest).

Author and Year	Drug Used and Method of Administration	Titanium Fixture Location	Follow-up Duration	Glucose Level of Diabetic Animals after Medication (mg/dL)	BIC (%) of Healthy Animals	Control BIC (%) (Untreated Diabetic)	Experimental BIC (%) (Treated Diabetic)	Other Outcomes of Interest
Guimarães et al., 2011 [31]	Aminoguanidine (intraperitoneal)	Implant in femur	4 weeks	Value provided as variation from baseline, no actual value given	-	-	-	Counter-torque values (N/cm) Healthy rats: 2.24 ± 0.58 Diabetic + medication: 2.5 ± 0.6 Diabetic rats: 1.18 ± 0.15
Aiala et al., 2013 [32]	Aminoguanidine (drug-loaded chitosan membrane)	Implant in femur	4 weeks	436.6 mg/dL (hyperglycemic)	-	-	-	Counter-torque values (N/cm) Healthy rats: 2.66 ± 0.91 Diabetic + medication: 1.8 ± 0.7 Diabetic rats: 1.26 ± 0.19
Kopman et al., 2005 [26]	Aminoguanidine (intraperitoneal)	Implant in femur	4 weeks	525.4 ± 46.9 mg/dL (hyperglycemic)	64.3 ± 3.7	22.2 ± 3.9	55.3 ± 6.1	Aminoguanidine augments osseointegration in diabetic rats
Bautista et al., 2019 [33]	Sitagliptin (oral)	Implant in tibia	4 weeks	590.7 ± 30.2 mg/dL (hyperglycemic)	-	-	-	Bone area fraction occupancy significantly lower in diabetic rats
Matsubara et al., 2001 [34]	Insulin (systemic delivery)	Implant in tibia	8 weeks	156 mg/dL (normoglycemic)	65.5	56.0	66.2	Lower BIC and bone volume in untreated diabetic rats
Zhang et al., 2021 [35]	Insulin (systemic delivery)	Implant in femur	12 weeks	Less than 180 mg/dL (normoglycemic)	61	20	Almost 50	Higher pull-out test values in insulin-treated rats; however, insulin alone does not adequately reverse effects of DM
de Molon et al., 2013 [27]	Insulin (systemic delivery)	Implant in tibia	16 weeks	75 ± 11 (normoglycemic)	63.37 ± 5.88	60.81 ± 6.83	66.97 ± 4.13	Bone area (%): Diabetic rats: 69.34 ± 5.00 Insulin-treated rats: 79.63 ± 4.97 Counter torque (N/cm): Diabetic rats: 12.91 ± 2.51 Insulin-treated rats: 17.10 ± 3.06
de Morais et al., 2009 [28]	Insulin (systemic delivery)	Implant in tibia	16 weeks	42 ± 8 (normoglycemic)	-	-	-	Digital subtraction radiography: Significantly lower peri-implant bone density and more bone loss in untreated diabetic rats
Kwon et al., 2005 [36]	Insulin (systemic delivery)	Implant in femur	16 weeks	HbA1c at 4.2 ± 0.1 (normoglycemic)	-	41.7 ± 2.3	61.4 ± 2.4	Untreated diabetic rats show gradual decrease in BIC over 4 months
Margonar et al., 2003 [37]	Insulin (systemic delivery)	Implant in tibia	12 weeks	99.2 ± 72.2 (normoglycemic)	-	-	-	Counter removal torque (N/cm): Diabetic rabbits: 22.0 ± 6.0 Insulin-treated rabbits: 25.2 ± 4.4
Siqueira et al., 2003 [38]	Insulin (systemic delivery)	Implant in tibia	21 days	295 ± 24 (hyperglycemic)	About 50	Less BIC (about 20)	Significantly improved BIC (about 50)	Need to control blood glucose for successful osseointegration
Fiorellini et al., 1999 [39]	Insulin (systemic delivery)	Implant in femur	4 weeks	145 ± 64.4 (8 mmol/L—slightly hyperglycemic)	50.4 ± 1.3	50.4 ± 1.3 (non-diabetic)	52.4 ± 0.0	Insulin administration for diabetic rats ameliorates bone formation to similar level with healthy controls
McCracken et al., 2006 [40]	Insulin (systemic delivery)	Implant in tibia	24 days	Less than 200 mg/dL (normoglycemic)	-	-	-	Bone volume (%): Diabetic rats: 22.6 ± 10 (significant) Insulin-treated rats: 17 ± 7
Yamazaki et al., 2020 [29]	Insulin (systemic delivery)	Implant in healed extraction site	8 weeks	Less than 100 mg/dL (normoglycemic)	-	-	-	Bone loss measured in micro-CT: Diabetic rats: 0.42 ± 0.05 mm Insulin-treated rats: 0.39 ± 0.10 mm (not significant)
Han et al., 2012 [41]	Insulin (local delivery)—FGIPM	Implant in tibia	8 weeks	383.4 ± 56.5 (hyperglycemic)	-	-	-	Counter torque (N/cm): Normal rats: 4.82 ± 0.5419 Diabetic rats: 3.07 ± 0.3266 Insulin-treated rats: 4.67 ± 0.3386 (significant)
Serrão et al., 2017 [42]	Metformin (oral)	Implant in tibia	4 weeks	154 ± 27 (normoglycemic)	70.17 ± 5.36	50.85 ± 6.26	48.58 ± 12.41	Increased levels of OPG, decreased RANKL/OPG ratio in medullary bone
Inouye et al., 2014 [43]	Metformin (oral)	Implant in healed extraction site	4 weeks	Fluctuates, 293.8 ± 28.0 mg/dL (hyperglycemic)	49.18 + 5.57	23.36 ± 6.44	31.57 ± 8.29	No significant difference in BIC at week 4 due to hyperglycemia at that point (fluctuation)
Hashiguchi et al., 2014 [44]	Voglibose (oral)	Implant in tibia	9 weeks	About 250 mg/dL (from graph) (hyperglycemic)	71.7 ± 11.2	55.640 ±16.578	40.999 ± 10.682	No significant difference in BIC and counter torque values
Zhou et al., 2015 [46]	Exenatide (subcutaneous)	Implant in femur	4 weeks, 8 weeks	Constant glucose concentration less than 288 mg/dL—unclear level of glycemia	-	-	-	At 4 weeks, incomplete peri-implant bone fill in diabetic controls, while woven bone and direct BIC surrounds implants with exenatide treatment
Liu et al., 2015 [45]	Exenatide (subcutaneous)	Implant in femur	4 weeks, 8 weeks	Constant glucose concentration less than 288 mg/dL—unclear level of glycemia	-	-	-	Higher bone density, less inflammatory cells, stronger expression of integrin α5β1 and fibronectin in exenatide group versus untreated diabetic rats
Wang et al., 2011 [47]	Insulin (local delivery)—insulin-containing PLGA microcapsules	Implant in tibiae	6 weeks	Not specified	66 ± 3	51 ± 3	58 ± 3	Significantly more BIC in insulin-treated group

BIC: bone-implant contact; FGIPM: fibrin gel loaded with insulin/polylactic-co-glycolic acid; HbA1c: glycated hemoglobin; micro-CT: microcomputed tomography; OPG: osteoprotegerin; PLGA: polylactic glycolic acid; RANKL: receptor activator of NF-κB (RANK) ligand; T1DM: type 1 diabetes mellitus; T2DM: type 2 diabetes mellitus.

**Table 4:** PICO research framework.

PICO Elements	Framework Details
Animal model representing patient population	Diabetic animals (T1DM and T2DM) that received titanium implants
Intervention reflecting clinical practice	Diabetic animals that received anti-diabetic treatment using approved medications in human clinical practice guidelines
Comparator without treatment or intervention	Diabetic animals that did not receive treatment for diabetes
Outcome (biological effect or mechanism)	Implant osseointegration outcomes (bone-implant contact, bone volume, bone area, counter-torque values, amount of bone loss, bone biomarkers level)

P: population, I: intervention, C: comparator, O: outcome of interest; T1DM: type 1 diabetes mellitus; T2DM: type 2 diabetes mellitus.

## Data Availability

The data used to support the findings of this study are included within the article or Supplementary Materials.

## Supplementary Materials

The following supporting information can be downloaded at: https://www.mdpi.com/article/10.3390/ph15121518/s1.
