# Supplementary material for: Effect of Anti-Diabetic Medications on Dental Implants: A Scoping Review of Animal Studies and Their Relevance to Humans
Source: Pharmaceuticals (Basel). 2022 Dec 5;15(12):1518. doi: 10.3390/ph15121518 (PMC9784528; doi:10.3390/ph15121518)
Supplement: Supplementary file 1 [file pharmaceuticals-15-01518-s001.zip › pharmaceuticals-2051688-supplementary.pdf]

Supplement A

Preferred Reporting Items for Systematic reviews and Meta-Analyses extension for Scoping Reviews (PRISMA-ScR) Checklist

SECTION	ITEM	PRISMA-ScR CHECKLIST ITEM	REPORTED ON PAGE #
**TITLE**			
Title	1	Identify the report as a scoping review.	1
**ABSTRACT**			
Structured summary	2	Provide a structured summary that includes (as applicable): background, objectives, eligibility criteria, sources of evidence, charting methods, results, and conclusions that relate to the review questions and objectives.	1
**INTRODUCTION**			
Rationale	3	Describe the rationale for the review in the context of what is already known. Explain why the review questions/objectives lend themselves to a scoping review approach.	2
Objectives	4	Provide an explicit statement of the questions and objectives being addressed with reference to their key elements (e.g., population or participants, concepts, and context) or other relevant key elements used to conceptualize the review questions and/or objectives.	2
**METHODS**			
Protocol and registration	5	Indicate whether a review protocol exists; state if and where it can be accessed (e.g., a Web address); and if available, provide registration information, including the registration number.	Nil
Eligibility criteria	6	Specify characteristics of the sources of evidence used as eligibility criteria (e.g., years considered, language, and publication status), and provide a rationale.	2-3
Information sources*	7	Describe all information sources in the search (e.g., databases with dates of coverage and contact with authors to identify additional sources), as well as the date the most recent search was executed.	2
Search	8	Present the full electronic search strategy for at least 1 database, including any limits used, such that it could be repeated.	Supplement B
Selection of sources of evidence†	9	State the process for selecting sources of evidence (i.e., screening and eligibility) included in the scoping review.	3
Data charting process‡	10	Describe the methods of charting data from the included sources of evidence (e.g., calibrated forms or forms that have been tested by the team before their use, and whether data charting was done independently or in duplicate) and any processes for obtaining and confirming data from investigators.	3
Data items	11	List and define all variables for which data were sought and any assumptions and simplifications made.	3
Critical appraisal of individual sources of evidence§	12	If done, provide a rationale for conducting a critical appraisal of included sources of evidence; describe the methods used and how this information was used in any data synthesis (if appropriate).	3, 4 and Supplement D
Synthesis of results	13	Describe the methods of handling and summarizing the data that were charted.	3

SECTION	ITEM	PRISMA-ScR CHECKLIST ITEM	REPORTED ON PAGE #
**RESULTS**			
Selection of sources of evidence	14	Give numbers of sources of evidence screened, assessed for eligibility, and included in the review, with reasons for exclusions at each stage, ideally using a flow diagram.	3
Characteristics of sources of evidence	15	For each source of evidence, present characteristics for which data were charted and provide the citations.	4, 7-8
Critical appraisal within sources of evidence	16	If done, present data on critical appraisal of included sources of evidence (see item 12).	6 and Supplement D
Results of individual sources of evidence	17	For each included source of evidence, present the relevant data that were charted that relate to the review questions and objectives.	4-5, 9-13
Synthesis of results	18	Summarize and/or present the charting results as they relate to the review questions and objectives.	4-5, 12-13
**DISCUSSION**			
Summary of evidence	19	Summarize the main results (including an overview of concepts, themes, and types of evidence available), link to the review questions and objectives, and consider the relevance to key groups.	13-15
Limitations	20	Discuss the limitations of the scoping review process.	15
Conclusions	21	Provide a general interpretation of the results with respect to the review questions and objectives, as well as potential implications and/or next steps.	16
**FUNDING**			
Funding	22	Describe sources of funding for the included sources of evidence, as well as sources of funding for the scoping review. Describe the role of the funders of the scoping review.	16

JBI = Joanna Briggs Institute; PRISMA-ScR = Preferred Reporting Items for Systematic reviews and Meta-Analyses extension for Scoping Reviews.
* Where *sources of evidence* (see second footnote) are compiled from, such as bibliographic databases, social media platforms, and Web sites.
† A more inclusive/heterogeneous term used to account for the different types of evidence or data sources (e.g., quantitative and/or qualitative research, expert opinion, and policy documents) that may be eligible in a scoping review as opposed to only studies. This is not to be confused with *information sources* (see first footnote).
‡ The frameworks by Arksey and O'Malley (6) and Levac and colleagues (7) and the JBI guidance (4, 5) refer to the process of data extraction in a scoping review as data charting.
§ The process of systematically examining research evidence to assess its validity, results, and relevance before using it to inform a decision. This term is used for items 12 and 19 instead of "risk of bias" (which is more applicable to systematic reviews of interventions) to include and acknowledge the various sources of evidence that may be used in a scoping review (e.g., quantitative and/or qualitative research, expert opinion, and policy document).

From: Tricco AC, Lillie E, Zarin W, O'Brien KK, Colquhoun H, Levac D, et al. PRISMA Extension for Scoping Reviews (PRISMAScR): Checklist and Explanation. Ann Intern Med. 2018;169:467–473. doi: 10.7326/M18-0850 [60].

Supplement B

Pubmed Search Protocol (210522)

Number	Keyword	Search results
**#1**	**((dental implant) OR (oral implant) OR (osseointegration) OR (titanium)) AND (diabetes)**	**1418**
	("dental implants"[MeSH Terms] OR ("dental"[All Fields] AND "implants"[All Fields]) OR "dental implants"[All Fields] OR ("dental"[All Fields] AND "implant"[All Fields]) OR "dental implant"[All Fields] OR (("mouth"[MeSH Terms] OR "mouth"[All Fields] OR "oral"[All Fields]) AND ("embryo implantation"[MeSH Terms] OR ("embryo"[All Fields] AND "implantation"[All Fields]) OR "embryo implantation"[All Fields] OR "implantation"[All Fields] OR "implant"[All Fields] OR "implant s"[All Fields] OR "implantability"[All Fields] OR "implantable"[All Fields] OR "implantables"[All Fields] OR "implantate"[All Fields] OR "implantated"[All Fields] OR "implantates"[All Fields] OR "implantations"[All Fields] OR "implanted"[All Fields] OR "implanter"[All Fields] OR "implanters"[All Fields] OR "implanting"[All Fields] OR "implantion"[All Fields] OR "implantitis"[All Fields] OR "implants"[All Fields])) OR ("osseointegrate"[All Fields] OR "osseointegrated"[All Fields] OR "osseointegrates"[All Fields] OR "osseointegrating"[All Fields] OR "osseointegration"[MeSH Terms] OR "osseointegration"[All Fields] OR "osseointegrative"[All Fields]) OR ("titanium"[MeSH Terms] OR "titanium"[All Fields] OR "titaniums"[All Fields])) AND ("diabete"[All Fields] OR "diabetes mellitus"[MeSH Terms] OR ("diabetes"[All Fields] AND "mellitus"[All Fields]) OR "diabetes mellitus"[All Fields] OR "diabetes"[All Fields] OR "diabetes insipidus"[MeSH Terms] OR ("diabetes"[All Fields] AND "insipidus"[All Fields]) OR "diabetes insipidus"[All Fields] OR "diabetic"[All Fields] OR "diabetics"[All Fields] OR "diabets"[All Fields])	
	**Translations**	
	**dental implant:** "dental implants"[MeSH Terms] OR ("dental"[All Fields] AND "implants"[All Fields]) OR "dental implants"[All Fields] OR ("dental"[All Fields] AND "implant"[All Fields]) OR "dental implant"[All Fields]	
	**oral:** "mouth"[MeSH Terms] OR "mouth"[All Fields] OR "oral"[All Fields]	
	**implant:** "embryo implantation"[MeSH Terms] OR ("embryo"[All Fields] AND "implantation"[All Fields]) OR "embryo implantation"[All Fields] OR "implantation"[All Fields] OR "implant"[All Fields] OR "implant's"[All Fields] OR "implantability"[All Fields] OR "implantable"[All Fields] OR "implantables"[All Fields] OR "implantate"[All Fields] OR "implantated"[All Fields] OR "implantates"[All Fields] OR "implantations"[All Fields] OR "implanted"[All Fields] OR "implanter"[All Fields] OR "implanters"[All Fields] OR "implanting"[All Fields] OR "implantion"[All Fields] OR "implantitis"[All Fields] OR "implants"[All Fields]	
	**osseointegration:** "osseointegrate"[All Fields] OR "osseointegrated"[All Fields] OR "osseointegrates"[All Fields] OR "osseointegrating"[All Fields] OR "osseointegration"[MeSH Terms] OR "osseointegration"[All Fields] OR "osseointegrative"[All Fields]	
	**titanium:** "titanium"[MeSH Terms] OR "titanium"[All Fields] OR "titanium's"[All Fields] OR "titaniums"[All Fields]	
	**diabetes:** "diabete"[All Fields] OR "diabetes mellitus"[MeSH Terms] OR ("diabetes"[All Fields] AND "mellitus"[All Fields]) OR "diabetes mellitus"[All Fields] OR "diabetes"[All Fields] OR "diabetes insipidus"[MeSH Terms] OR ("diabetes"[All	

Fields] AND "insipidus"[All Fields]) OR "diabetes insipidus"[All Fields] OR "diabetic"[All Fields] OR "diabetics"[All Fields] OR "diabets"[All Fields]

**#2**	**((animal) OR (pre-clinical) OR (experimental)) AND (#1)**	**417**

("animals"[MeSH Terms:noexp] OR "animal"[All Fields] OR "pre-clinical"[All Fields] OR ("experimental"[All Fields] OR "experimentally"[All Fields] OR "experimentals"[All Fields] OR "experimentation"[All Fields] OR "experimentations"[All Fields] OR "experimenter"[All Fields] OR "experimenter s"[All Fields] OR "experimenters"[All Fields])) AND (("dental implants"[MeSH Terms] OR ("dental"[All Fields] AND "implants"[All Fields]) OR "dental implants"[All Fields] OR ("dental"[All Fields] AND "implant"[All Fields]) OR "dental implant"[All Fields] OR (("mouth"[MeSH Terms] OR "mouth"[All Fields] OR "oral"[All Fields]) AND ("embryo implantation"[MeSH Terms] OR ("embryo"[All Fields] AND "implantation"[All Fields]) OR "embryo implantation"[All Fields] OR "implantation"[All Fields] OR "implant"[All Fields] OR "implant s"[All Fields] OR "implantability"[All Fields] OR "implantable"[All Fields] OR "implantables"[All Fields] OR "implantate"[All Fields] OR "implantated"[All Fields] OR "implantates"[All Fields] OR "implantations"[All Fields] OR "implanted"[All Fields] OR "implanter"[All Fields] OR "implanters"[All Fields] OR "implanting"[All Fields] OR "implantion"[All Fields] OR "implantitis"[All Fields] OR "implants"[All Fields])) OR ("osseointegrate"[All Fields] OR "osseointegrated"[All Fields] OR "osseointegrates"[All Fields] OR "osseointegrating"[All Fields] OR "osseointegration"[MeSH Terms] OR "osseointegration"[All Fields] OR "osseointegrative"[All Fields]) OR ("titanium"[MeSH Terms] OR "titanium"[All Fields] OR "titaniums"[All Fields])) AND ("diabete"[All Fields] OR "diabetes mellitus"[MeSH Terms] OR ("diabetes"[All Fields] AND "mellitus"[All Fields]) OR "diabetes mellitus"[All Fields] OR "diabetes"[All Fields] OR "diabetes insipidus"[MeSH Terms] OR ("diabetes"[All Fields] AND "insipidus"[All Fields]) OR "diabetes insipidus"[All Fields] OR "diabetic"[All Fields] OR "diabetics"[All Fields] OR "diabets"[All Fields]))

Translations

animal: "animals"[MeSH Terms:noexp] OR animal[All Fields]

experimental: "experimental"[All Fields] OR "experimentally"[All Fields] OR "experimentals"[All Fields] OR "experimentation"[All Fields] OR "experimentations"[All Fields] OR "experimenter"[All Fields] OR "experimenter's"[All Fields] OR "experimenters"[All Fields]

dental implant: "dental implants"[MeSH Terms] OR ("dental"[All Fields] AND "implants"[All Fields]) OR "dental implants"[All Fields] OR ("dental"[All Fields] AND "implant"[All Fields]) OR "dental implant"[All Fields]

oral: "mouth"[MeSH Terms] OR "mouth"[All Fields] OR "oral"[All Fields]

implant: "embryo implantation"[MeSH Terms] OR ("embryo"[All Fields] AND "implantation"[All Fields]) OR "embryo implantation"[All Fields] OR "implantation"[All Fields] OR "implant"[All Fields] OR "implant's"[All Fields] OR "implantability"[All Fields] OR "implantable"[All Fields] OR "implantables"[All Fields] OR "implantate"[All Fields] OR "implantated"[All Fields] OR "implantates"[All Fields] OR "implantations"[All Fields] OR "implanted"[All Fields] OR "implanter"[All Fields] OR "implanters"[All Fields] OR "implanting"[All Fields] OR "implantion"[All Fields] OR "implantitis"[All Fields] OR "implants"[All Fields]

osseointegration: "osseointegrate"[All Fields] OR "osseointegrated"[All Fields] OR "osseointegrates"[All Fields] OR "osseointegrating"[All Fields] OR

"osseointegration"[MeSH Terms] OR "osseointegration"[All Fields] OR "osseointegrative"[All Fields]

titanium: "titanium"[MeSH Terms] OR "titanium"[All Fields] OR "titanium's"[All Fields] OR "titaniums"[All Fields]

diabetes: "diabete"[All Fields] OR "diabetes mellitus"[MeSH Terms] OR ("diabetes"[All Fields] AND "mellitus"[All Fields]) OR "diabetes mellitus"[All Fields] OR "diabetes"[All Fields] OR "diabetes insipidus"[MeSH Terms] OR ("diabetes"[All Fields] AND "insipidus"[All Fields]) OR "diabetes insipidus"[All Fields] OR "diabetic"[All Fields] OR "diabetics"[All Fields] OR "diabets"[All Fields]

**#3**	**((hypoglycemic agents) OR (anti-diabetic medication) OR (insulin)) AND (#2)**	**149**

("hypoglycaemic agents"[All Fields] OR "hypoglycemic agents"[Pharmacological Action] OR "hypoglycemic agents"[MeSH Terms] OR ("hypoglycemic"[All Fields] AND "agents"[All Fields]) OR "hypoglycemic agents"[All Fields] OR ("anti-diabetic"[All Fields] AND ("medic"[All Fields] OR "medical"[All Fields] OR "medicalization"[MeSH Terms] OR "medicalization"[All Fields] OR "medicalizations"[All Fields] OR "medicalize"[All Fields] OR "medicalized"[All Fields] OR "medicalizes"[All Fields] OR "medicalizing"[All Fields] OR "medically"[All Fields] OR "medicals"[All Fields] OR "medicated"[All Fields] OR "medication s"[All Fields] OR "medics"[All Fields] OR "pharmaceutical preparations"[MeSH Terms] OR ("pharmaceutical"[All Fields] AND "preparations"[All Fields]) OR "pharmaceutical preparations"[All Fields] OR "medication"[All Fields] OR "medications"[All Fields])) OR ("insulin"[MeSH Terms] OR "insulin"[All Fields] OR "insulin s"[All Fields] OR "insuline"[All Fields] OR "insulinic"[All Fields] OR "insulinization"[All Fields] OR "insulinized"[All Fields] OR "insulins"[MeSH Terms] OR "insulins"[All Fields])) AND (("animals"[MeSH Terms:noexp] OR "animal"[All Fields] OR "pre-clinical"[All Fields] OR ("experimental"[All Fields] OR "experimentally"[All Fields] OR "experimentals"[All Fields] OR "experimentation"[All Fields] OR "experimentations"[All Fields] OR "experimenter"[All Fields] OR "experimenter s"[All Fields] OR "experimenters"[All Fields])) AND (("dental implants"[MeSH Terms] OR ("dental"[All Fields] AND "implants"[All Fields]) OR "dental implants"[All Fields] OR ("dental"[All Fields] AND "implant"[All Fields]) OR "dental implant"[All Fields] OR (("mouth"[MeSH Terms] OR "mouth"[All Fields] OR "oral"[All Fields]) AND ("embryo implantation"[MeSH Terms] OR ("embryo"[All Fields] AND "implantation"[All Fields]) OR "embryo implantation"[All Fields] OR "implantation"[All Fields] OR "implant"[All Fields] OR "implant s"[All Fields] OR "implantability"[All Fields] OR "implantable"[All Fields] OR "implantables"[All Fields] OR "implantate"[All Fields] OR "implantated"[All Fields] OR "implantates"[All Fields] OR "implantations"[All Fields] OR "implanted"[All Fields] OR "implanter"[All Fields] OR "implanters"[All Fields] OR "implanting"[All Fields] OR "implantion"[All Fields] OR "implantitis"[All Fields] OR "implants"[All Fields])) OR ("osseointegrate"[All Fields] OR "osseointegrated"[All Fields] OR "osseointegrates"[All Fields] OR "osseointegrating"[All Fields] OR "osseointegration"[MeSH Terms] OR "osseointegration"[All Fields] OR "osseointegrative"[All Fields]) OR ("titanium"[MeSH Terms] OR "titanium"[All Fields] OR "titaniums"[All Fields])) AND ("diabete"[All Fields] OR "diabetes mellitus"[MeSH Terms] OR ("diabetes"[All Fields] AND "mellitus"[All Fields]) OR "diabetes mellitus"[All Fields] OR "diabetes"[All Fields] OR "diabetes insipidus"[MeSH Terms] OR ("diabetes"[All Fields] AND "insipidus"[All Fields]) OR "diabetes insipidus"[All Fields] OR "diabetic"[All Fields] OR "diabetics"[All Fields] OR "diabets"[All Fields])))

Translations

hypoglycemic agents: "hypoglycaemic agents"[All Fields] OR "hypoglycemic agents"[Pharmacological Action] OR "hypoglycemic agents"[MeSH Terms] OR ("hypoglycemic"[All Fields] AND "agents"[All Fields]) OR "hypoglycemic agents"[All Fields]

medication: "medic"[All Fields] OR "medical"[All Fields] OR "medicalization"[MeSH Terms] OR "medicalization"[All Fields] OR "medicalizations"[All Fields] OR "medicalize"[All Fields] OR "medicalized"[All Fields] OR "medicalizes"[All Fields] OR "medicalizing"[All Fields] OR "medically"[All Fields] OR "medicals"[All Fields] OR "medicated"[All Fields] OR "medication's"[All Fields] OR "medics"[All Fields] OR "pharmaceutical preparations"[MeSH Terms] OR ("pharmaceutical"[All Fields] AND "preparations"[All Fields]) OR "pharmaceutical preparations"[All Fields] OR "medication"[All Fields] OR "medications"[All Fields]

insulin: "insulin"[MeSH Terms] OR "insulin"[All Fields] OR "insulin's"[All Fields] OR "insuline"[All Fields] OR "insulinic"[All Fields] OR "insulinization"[All Fields] OR "insulinized"[All Fields] OR "insulins"[MeSH Terms] OR "insulins"[All Fields]

animal: "animals"[MeSH Terms:noexp] OR animal[All Fields]

experimental: "experimental"[All Fields] OR "experimentally"[All Fields] OR "experimentals"[All Fields] OR "experimentation"[All Fields] OR "experimentations"[All Fields] OR "experimenter"[All Fields] OR "experimenter's"[All Fields] OR "experimenters"[All Fields]

dental implant: "dental implants"[MeSH Terms] OR ("dental"[All Fields] AND "implants"[All Fields]) OR "dental implants"[All Fields] OR ("dental"[All Fields] AND "implant"[All Fields]) OR "dental implant"[All Fields]

oral: "mouth"[MeSH Terms] OR "mouth"[All Fields] OR "oral"[All Fields]

implant: "embryo implantation"[MeSH Terms] OR ("embryo"[All Fields] AND "implantation"[All Fields]) OR "embryo implantation"[All Fields] OR "implantation"[All Fields] OR "implant"[All Fields] OR "implant's"[All Fields] OR "implantability"[All Fields] OR "implantable"[All Fields] OR "implantables"[All Fields] OR "implantate"[All Fields] OR "implantated"[All Fields] OR "implantates"[All Fields] OR "implantations"[All Fields] OR "implanted"[All Fields] OR "implanter"[All Fields] OR "implanters"[All Fields] OR "implanting"[All Fields] OR "implantion"[All Fields] OR "implantitis"[All Fields] OR "implants"[All Fields]

osseointegration: "osseointegrate"[All Fields] OR "osseointegrated"[All Fields] OR "osseointegrates"[All Fields] OR "osseointegrating"[All Fields] OR "osseointegration"[MeSH Terms] OR "osseointegration"[All Fields] OR "osseointegrative"[All Fields]

titanium: "titanium"[MeSH Terms] OR "titanium"[All Fields] OR "titanium's"[All Fields] OR "titaniums"[All Fields]

diabetes: "diabete"[All Fields] OR "diabetes mellitus"[MeSH Terms] OR ("diabetes"[All Fields] AND "mellitus"[All Fields]) OR "diabetes mellitus"[All Fields] OR "diabetes"[All Fields] OR "diabetes insipidus"[MeSH Terms] OR ("diabetes"[All Fields] AND "insipidus"[All Fields]) OR "diabetes insipidus"[All Fields] OR "diabetic"[All Fields] OR "diabetics"[All Fields] OR "diabets"[All Fields]

**#4**	**((dental implants OR oral implants OR titanium OR osseointegration) AND (diabetes)) AND ((animal OR pre-clinical OR experimental)) AND (medication OR hyperglycaemic agents OR insulin)**	**245**

("dental implants"[MeSH Terms] OR ("dental"[All Fields] AND "implants"[All Fields]) OR "dental implants"[All Fields] OR (("mouth"[MeSH Terms] OR "mouth"[All Fields] OR "oral"[All Fields]) AND ("embryo implantation"[MeSH Terms] OR ("embryo"[All Fields] AND "implantation"[All Fields]) OR "embryo implantation"[All Fields] OR "implantation"[All Fields] OR "implant"[All Fields] OR "implant s"[All Fields] OR "implantability"[All Fields] OR "implantable"[All Fields] OR "implantables"[All Fields] OR "implantate"[All Fields] OR "implantated"[All Fields] OR "implantates"[All Fields] OR "implantations"[All Fields] OR "implanted"[All Fields] OR "implanter"[All Fields] OR "implanters"[All Fields] OR "implanting"[All Fields] OR "implantion"[All Fields] OR "implantitis"[All Fields] OR "implants"[All Fields])) OR ("titanium"[MeSH Terms] OR "titanium"[All Fields] OR "titaniums"[All Fields]) OR ("osseointegrate"[All Fields] OR "osseointegrated"[All Fields] OR "osseointegrates"[All Fields] OR "osseointegrating"[All Fields] OR "osseointegration"[MeSH Terms] OR "osseointegration"[All Fields] OR "osseointegrative"[All Fields])) AND ("diabete"[All Fields] OR "diabetes mellitus"[MeSH Terms] OR ("diabetes"[All Fields] AND "mellitus"[All Fields]) OR "diabetes mellitus"[All Fields] OR "diabetes"[All Fields] OR "diabetes insipidus"[MeSH Terms] OR ("diabetes"[All Fields] AND "insipidus"[All Fields]) OR "diabetes insipidus"[All Fields] OR "diabetic"[All Fields] OR "diabetics"[All Fields] OR "diabets"[All Fields]) AND ("animals"[MeSH Terms:noexp] OR "animal"[All Fields] OR "pre-clinical"[All Fields] OR ("experimental"[All Fields] OR "experimentally"[All Fields] OR "experimentals"[All Fields] OR "experimentation"[All Fields] OR "experimentations"[All Fields] OR "experimenter"[All Fields] OR "experimenter s"[All Fields] OR "experimenters"[All Fields])) AND ("medic"[All Fields] OR "medical"[All Fields] OR "medicalization"[MeSH Terms] OR "medicalization"[All Fields] OR "medicalizations"[All Fields] OR "medicalize"[All Fields] OR "medicalized"[All Fields] OR "medicalizes"[All Fields] OR "medicalizing"[All Fields] OR "medically"[All Fields] OR "medicals"[All Fields] OR "medicated"[All Fields] OR "medication s"[All Fields] OR "medics"[All Fields] OR "pharmaceutical preparations"[MeSH Terms] OR ("pharmaceutical"[All Fields] AND "preparations"[All Fields]) OR "pharmaceutical preparations"[All Fields] OR "medication"[All Fields] OR "medications"[All Fields] OR (("hyperglycaemic"[All Fields] OR "hyperglycaemics"[All Fields] OR "hyperglycemic"[All Fields] OR "hyperglycemics"[All Fields]) AND ("agent"[All Fields] OR "agents"[All Fields])) OR ("insulin"[MeSH Terms] OR "insulin"[All Fields] OR "insulin s"[All Fields] OR "insuline"[All Fields] OR "insulinic"[All Fields] OR "insulinization"[All Fields] OR "insulinized"[All Fields] OR "insulins"[MeSH Terms] OR "insulins"[All Fields]))

Translations

dental implants: "dental implants"[MeSH Terms] OR ("dental"[All Fields] AND "implants"[All Fields]) OR "dental implants"[All Fields]

oral: "mouth"[MeSH Terms] OR "mouth"[All Fields] OR "oral"[All Fields]

implants: "embryo implantation"[MeSH Terms] OR ("embryo"[All Fields] AND "implantation"[All Fields]) OR "embryo implantation"[All Fields] OR "implantation"[All Fields] OR "implant"[All Fields] OR "implant's"[All Fields] OR "implantability"[All Fields] OR "implantable"[All Fields] OR "implantables"[All Fields] OR "implantate"[All Fields] OR "implantated"[All Fields] OR "implantates"[All Fields] OR "implantations"[All Fields] OR "implanted"[All Fields] OR "implanter"[All Fields] OR "implanters"[All Fields] OR "implanting"[All Fields] OR "implantion"[All Fields] OR "implantitis"[All Fields] OR "implants"[All Fields]

titanium: "titanium"[MeSH Terms] OR "titanium"[All Fields] OR "titanium's"[All Fields] OR "titaniums"[All Fields]

osseointegration: "osseointegrate"[All Fields] OR "osseointegrated"[All Fields] OR "osseointegrates"[All Fields] OR "osseointegrating"[All Fields] OR "osseointegration"[MeSH Terms] OR "osseointegration"[All Fields] OR "osseointegrative"[All Fields]

diabetes: "diabete"[All Fields] OR "diabetes mellitus"[MeSH Terms] OR ("diabetes"[All Fields] AND "mellitus"[All Fields]) OR "diabetes mellitus"[All Fields] OR "diabetes"[All Fields] OR "diabetes insipidus"[MeSH Terms] OR ("diabetes"[All Fields] AND "insipidus"[All Fields]) OR "diabetes insipidus"[All Fields] OR "diabetic"[All Fields] OR "diabetics"[All Fields] OR "diabets"[All Fields]

animal: "animals"[MeSH Terms:noexp] OR animal[All Fields]

experimental: "experimental"[All Fields] OR "experimentally"[All Fields] OR "experimentals"[All Fields] OR "experimentation"[All Fields] OR "experimentations"[All Fields] OR "experimenter"[All Fields] OR "experimenter's"[All Fields] OR "experimenters"[All Fields]

medication: "medic"[All Fields] OR "medical"[All Fields] OR "medicalization"[MeSH Terms] OR "medicalization"[All Fields] OR "medicalizations"[All Fields] OR "medicalize"[All Fields] OR "medicalized"[All Fields] OR "medicalizes"[All Fields] OR "medicalizing"[All Fields] OR "medically"[All Fields] OR "medicals"[All Fields] OR "medicated"[All Fields] OR "medication's"[All Fields] OR "medics"[All Fields] OR "pharmaceutical preparations"[MeSH Terms] OR ("pharmaceutical"[All Fields] AND "preparations"[All Fields]) OR "pharmaceutical preparations"[All Fields] OR "medication"[All Fields] OR "medications"[All Fields]

hyperglycaemic: "hyperglycaemic"[All Fields] OR "hyperglycaemics"[All Fields] OR "hyperglycemic"[All Fields] OR "hyperglycemics"[All Fields]

agents: "agent"[All Fields] OR "agents"[All Fields]

insulin: "insulin"[MeSH Terms] OR "insulin"[All Fields] OR "insulin's"[All Fields] OR "insuline"[All Fields] OR "insulinic"[All Fields] OR "insulinization"[All Fields] OR "insulinized"[All Fields] OR "insulins"[MeSH Terms] OR "insulins"[All Fields]

Cochrane Library Search Protocol - Cochrane Central Register of Controlled Trials (210522)

Number	Keyword (Word variations have been searched)	Search results
#1	(dental implants):ti,ab,kw OR (oral implants):ti,ab,kw OR (titanium):ti,ab,kw OR (osseointegration):ti,ab,kw	9343
#2	(diabetes):ti,ab,kw	105844
#3	(preclinical):ti,ab,kw OR (animal):ti,ab,kw OR (experimental):ti,ab,kw	124631
#4	#1 AND #2 AND #3	35

EBSCOhost (Dentistry & Oral Sciences Source) (21052022)

Number	Keyword	Search results
S1	oral implant OR dental implants OR titanium OR osseointegration	74,681
S2	diabetes OR hypoglycemic drugs OR hypoglycemic agents OR insulin OR antidiabetic medication OR antidiabetic drugs	31,496
S3	animals OR experimental study OR preclinical OR experimental design	67,918
S4	S1 AND S2 AND S3	3,879
S5	S4 NOT review	574
S6	S5 (apply Filter "Source Type – Academic Journals")	560

Supplement C

List of excluded studies with reasoning

No.	Study Title	Reason for exclusion
1	Goodman, W. G., & Hori, M. T. (1984). Diminished bone formation in experimental diabetes: relationship to osteoid maturation and mineralization. Diabetes, 33(9), 825-831 [24].	No dental implant placed
2	Wang X, Lu Q, Yu DS, et al. Asiatic acid mitigates hyperglycemia and reduces islet fibrosis in Goto-Kakizaki rat, a spontaneous type 2 diabetic animal model. Chin J Nat Med. 2015;13(7):529-534 [14].	Use of supplement or off-label drug for diabetic treatment
3	Rybaczek T, Tangl S, Dobsak T, Gruber R, Kuchler U. The Effect of Parathyroid Hormone on Osseointegration in Insulin-Treated Diabetic Rats. *Implant Dent*. 2015;24(4):392-396 [15].	Use of supplement or off-label drug for diabetic treatment
4	Wang F, Song Y liang, Li C xia, et al. Sustained release of insulin-like growth factor-1 from poly(lactide-co-glycolide) microspheres improves osseointegration of dental implants in type 2 diabetic rats. *Eur J Pharmacol*. 2010;640(1-3):226-232 [47].	Use of supplement or off-label drug for diabetic treatment
5	Cirano FR, Pimentel SP, Casati MZ, et al. Effect of curcumin on bone tissue in the diabetic rat: repair of peri-implant and critical-sized defects. *Int J Oral Maxillofac Surg*. 2018;47(11):1495-1503 [17].	Use of supplement or off-label drug for diabetic treatment
6	Corrêa MG, Ribeiro FV, Pimentel SP, et al. Impact of resveratrol in the reduction of the harmful effect of diabetes on peri-implant bone repair: bone-related gene expression, counter-torque and micro-CT analysis in rats. Acta Odontol Scand. Published online 2020:1-8 [18].	Use of supplement or off-label drug for diabetic treatment
7	Shao, J., Liu, S., Zheng, X., Chen, J., Li, L., & Zhu, Z. (2021). Berberine promotes peri-implant osteogenesis in diabetic rats by ROS-mediated IRS-1 pathway. BioFactors, 47(1), 80-92.	Use of supplement or off-label drug for diabetic treatment
8	Takeshita F, Murai K, Iyama S, Ayukawa Y, Suetsugu T. Uncontrolled Diabetes Hinders Bone Formation Around Titanium Implants in Rat Tibiae. A Light and Fluorescence Microscopy, and Image Processing Study. J Periodontol. 1998;69(3):314-320 [20].	No treatment given for diabetic animals
9	Zhou, Wenjuan, et al. "The Influence of Type 2 Diabetes Mellitus on the Osseointegration of Titanium Implants With Different Surface Modifications—A Histomorphometric Study in High-Fat Diet/Low-Dose Streptozotocin–Treated Rats." Implant dentistry 28.1 (2019): 11-19 [21].	No treatment given for diabetic animals
10	Saito, Natsumi, et al. "Impaired dental implant osseointegration in rat with streptozotocin-induced diabetes." Journal of Periodontal Research (2022) [22].	No treatment given for diabetic animals
11	Sun, Rongxin, et al. "Effects of metformin on the osteogenesis of alveolar BMSCs from diabetic patients and implant osseointegration in rats." Oral Diseases (2021) [23].	Anti-diabetic drug given to healthy animals
12	Lin, Jiating, et al. "Metformin promotes the osseointegration of titanium implants under osteoporotic conditions by regulating BMSCs autophagy, and osteogenic differentiation." Biochemical and Biophysical Research Communications 531.2 (2020): 228-235.	Anti-diabetic drug given to osteoporotic animals (non-diabetic model)
13	Zhou, Wenjuan, et al. "Effects of Delayed Release Microsphere of Exenatide on Peri-Implant Bone Response of Diabetic Rats." Dentistry 5.335 (2015): 2161-1122.	Less than 10 implants in study design

Supplement D

Risk of Bias (RoB) assessment using SYRCLE tool

Domains	Selection Bias			Performance Bias		Detection Bias		Attrition Bias	Reporting Bias	Other sources of Bias	Summary
	1	2	3	4	5	6	7	8	9	10	
Author and year	Sequence generation	Baseline characteristics	Allocation concealment	Random housing	Blinded investigator	Random outcome assessment	Blinding of outcome assessor	Incomplete outcome data	Selective outcome reporting	Other sources of bias	
(Serrão et al., 2017 [42])	+	+	? Method of randomization not mentioned	? Rats kept alone in cage with feed and water	+	-	"Same trained, calibrated and blinded examiner (D.F.C)" - inappropriate blinding	+	-	- Residual hyperglycemia even though treated with metformin	?
(Inouye et al., 2014 [43])	-	- At the time of extraction and 1st week, metformin-treated rats had hyperglycemia	-	-	-	-	-	+	+	- Failed to control hyperglycemia throughout the experiment, no randomization, blinding or calibration mentioned	-
(Guimarães et al. 2011 [31])	+	? Data regarding baseline blood glucose was described as variation rather than actual value	-	-	-	-	-	+	+	Glycemia level varied widely from 250mg/dL to 500mg/dL within the diabetic group	?
(Aiala et al. 2013 [32])	+ randomly distributed by lot	+ Equal number of animals, weight and glucose levels in each group	? Method of randomization not mentioned	? Housed in department	-	-	-	+	+	No actual treatment of diabetes was given, hence animals were hyperglycemic throughout the experiment	?

(Kopman et al., 2005 [26])	+	Baseline characteristics were the same, but at Day 0 and throughout the experiment, aminoguanidine-treated rats had hyperclycemia	? Method of randomization not mentioned	-	-	+ blood samples were taken from three random animals in each grou	"measured by three calibrated and blinded examiners" +	+	+	- Hyperglycemia even though treated with aminoguanidine	-
(Hashiguchi et al., 2014 [24])	-	+ Same baseline characteristics	-	-	-	-	-	+	+	- Failed to control hyperglycemia throughout the experiment, no randomization, blinding or calibration mentioned	- no difference in BIC between Diabetic or treated animals, but unable to determine if this is due to hyperglycemia or voglibose
(Liu et al., 2015 [45])	-	+ Same baseline characteristics	-	-	-	-	-	+	+	? Blood glucose controlled at a constant level using exenatide. However the level is still hyperglycemic (above 10mmol/L)	?
(Zhou et al., 2015 [46])	-	+ Same baseline characteristics	-	-	-	-	-	+	+	? Blood glucose controlled at a constant level using exenatide. However the level is still hyperglycemic (above 10mmol/L)	?
(Bautista et al., 2019 [33])	-	+ Same baseline characteristics	-	-	-	-	+ single-blind	+	+	- Error in methodology, failure to induce T2DM, sitagliptin is meant for T2DM treatment	-
(Matsubara et al., 2001 [34])	-	+ Same baseline characteristics	-	-	-	-	-	+	+	No other obvious source of bias	?

(Zhang et al., 2021 [35])	+ randomly divided	+ Same baseline characteristics	? Method of randomization not mentioned	-	-	-	-	+	+	No other obvious source of bias	?
(de Molon et al., 2013)	+ randomly divided	+ Same baseline characteristics	? Method of randomization not mentioned	-	-	+	+	+	+	No other obvious source of bias	+
(de Morais et al., 2009 [28])	+ randomly divided	+ Same baseline characteristics	? Method of randomization not mentioned	-	-	+	+	+	+	No other obvious source of bias	+
(Kwon et al., 2005 [36])	-	- not mentioned	-	-	-	-	+ 3 masked calibrated examiners	+	+	Small sample size, only 1 animal per group (3 implants each)	?
(Margonar et al., 2003 [37])	+ randomly divided	+ Same baseline characteristics	? Method of randomization not mentioned	- individual housing	-	-	-	+	+	No other obvious source of bias	?
(Siqueira et al., 2003 [38])	- allocation by availability of intervention	+ Same baseline characteristics, insulin started for diabetic animals at day 10 after implant insertion	-	-	-	-	-	+	+	- Hyperglycemia even though treated with insulin	?
(Fiorellini et al., 1999 [39])	-	+ Same baseline characteristics	-	-	-	-	-	+	+	No other obvious source of bias	?
(McCracken et al., 2006 [40])	- allocation by availability of intervention	+ Same baseline characteristics	-	-	-	-	-	-	+	Change in methodology midway due to loss of insulin treatment. Calculations adjusted for missing and incomplete data	-

(Han et al. 2012 [41])	-	+ Same baseline characteristics	-	-	-	-	-	+	+	No actual treatment of diabetes was given (local only), hence animals were hyperglycemic throughout the experiment	-
(Wang et al. 2011 [47])	-	+ Same baseline characteristics, different type of rat as control group	-	-	-	-	-	+	+	? No measurement of systemic glucose level	?
Yamazaki et al. 2020 [29]	+	+ Same baseline characteristics	+ random permutations	-	-	-	+	+	+	None, uses ARRIVE guideline for reporting	+
